# The role of PDIA3 in myogenesis during muscle regeneration

**DOI:** 10.1038/s12276-019-0368-2

**Published:** 2020-01-20

**Authors:** Chao Wang, Yuanjiao Zhu, Dan Wu, Zien Wang, Xiaoli Xu, Yan Shi, Gang Yang, Yongming Yu, Xi Peng

**Affiliations:** 1 State Key Laboratory of Trauma, Burns and Combined Injury, Clinical Medical Research Center, Southwest Hospital, Third Military Medical University (The Army Medical University), Chongqing, 400038 China; 2 Department of Burns and Plastic Surgery, Chengdu Second People’s Hospital, Chengdu, 610011 China; 3 Department of Burns, Union Hospital, Fujian Medical University, Fuzhou, 350001 China; 4 Department of Geriatric Medicine, Chengdu Second People’s Hospital, Chengdu, 610011 China; 5 Shriners Burns Hospital, Massachusetts General Hospital, Harvard Medical School, Boston, MA 02114 USA

**Keywords:** Stem-cell research, Muscle stem cells

## Abstract

Beta 3 (β3) integrin plays an important role in the initiation of myogenesis in adult muscle. Protein disulfide isomerases (PDIs) can activate β3 integrin in various cells to promote cell migration, adhesion and fusion. However, the effect of PDIs on myogenesis during muscle regeneration has not been elucidated. Here, we report that PDIA3 expression is induced in regenerating myofibers. The inhibition of PDIA3 in muscle injuries in mice disrupts myoblast differentiation, impairs muscle regeneration, and ultimately aggravates muscle damage. Moreover, PDIA3 expression is upregulated and observed on the cell surfaces of myoblasts during differentiation and fusion. The inhibition of extracellular PDIA3 with an anti-PDIA3 monoclonal antibody attenuates β3 integrin/AKT/mTOR signal activity, inhibits myoblast differentiation, and blocks the fusion of myoblasts. Thus, PDIA3 may be a mediator of myoblast differentiation and fusion during muscle regeneration.

## Muscle development: Regeneration after injury

A regulatory protein secreted into the extracellular medium helps promote formation of new skeletal muscle tissue after injury. This regenerative process is enabled by muscle cell progenitors called satellite cells, which respond to damage by developing into myoblasts that subsequently fuse into muscle fibers. Researchers led by Xi Peng of the Third Military Medical University in Chongqing, China, have identified the protein PDIA3 as an important intermediary in this process. After chemically inducing muscle damage in mice, the researchers noted that tissue regeneration was associated with a sharp increase in PDIA3 expression. They subsequently linked PDIA3 with activation of a signaling pathway that drives muscle fiber formation and fusion, and demonstrated that inhibition of this protein impeded healing. PDIA3 was not strongly expressed in satellite cells, indicating that it acts relatively late in the maturation process.

## Introduction

Skeletal muscle is the major component of the human locomotion system. It is also recognized as a metabolic organ that supplies amino acids and utilizes glucose and fatty acids^1–3^. Skeletal muscle damage is a frequent clinical problem that is usually secondary to burn injury, trauma, and myopathy caused by conditions such as Duchenne muscular dystrophy (DMD)^4–8^. Muscle regeneration deficiency causes injuries to heal slowly and improperly, resulting in muscle fibrosis and the loss of normal muscle function^9,10^. In contrast, efficient muscle regeneration following muscle injury is crucial for restoring the physiological function of skeletal muscle. Therefore, skeletal muscle regeneration is an essential process required for maintaining the contractile function of muscle and whole-body energy metabolism^10,11^.

The regenerative capacity of skeletal muscle mainly depends on satellite cells (SCs), which are normally quiescent. After muscle injury, these cells are induced to undergo myogenesis through coordinated myogenic marker expression and cell migration and fusion and ultimately form new myofibers^12,13^. However, the mechanisms by which SCs sense muscle damage and undergo myogenesis in an orderly manner have not been fully elucidated.

Integrins are a family of transmembrane receptors that mediate cell–cell and cell–extracellular matrix (ECM) adhesion^14^. In addition, integrins can cooperate with multiple growth factors and generate many intracellular signals to regulate cell proliferation, differentiation, and fusion. Integrins are heterodimers of two subunits, α and β. Integrins in mammals have 24 α and 9 β subunits^15–17^. In skeletal muscle, the integrin αVβ3 is crucial for the maintenance of muscle integrity, as mice with defects in β3 integrin exhibit the failure of myogenesis during muscle regeneration. In addition, αVβ3 integrin has been demonstrated to mediate myogenesis through promoting myogenic gene expression and the migration and fusion of SCs^18^.

Protein disulfide isomerases (PDIs) are multifunctional chaperones that regulate multiple protein functions via the oxidation, reduction, and isomerization of protein disulfide bonds. Most PDI family members (PDIs) are localized within the endoplasmic reticulum (ER); however, some PDI family members can escape from the ER and be secreted into the ECM or translocate to the cell surface, such as PDIA1 and PDIA3^19^.

Extracellular PDIA1 and PDIA3 have recently been identified as two activators of αVβ3 integrin through isomerization of disulfide bonds in integrin β3 subunits. The interaction between PDI and β3 integrin plays a key role in mediating multiple biological processes, including platelet adhesion^20^, neutrophil migration^21^, T cell migration^22^, and gamete fusion^23^. Blockade of either PDIA1 or PDIA3 at the cell surface causes β3 integrin inactivation and then impairs the cellular capacities for migration, adhesion, and fusion, which are all basic cell behaviors involved in myogenesis during muscle regeneration^18,20–22^. However, whether the mediation of the activation of β3 integrin by PDIA1 and PDIA3 plays a role in myogenesis is unknown.

Here, we hypothesized that PDI family members play a major regulatory role in myogenesis through their interactions with β3 integrin. In the present study, we revealed for the first time that PDIA3 is persistently expressed in neonatal myofibers during muscle regeneration and that it facilitates myogenesis via mediating myogenic differentiation and fusion.

## Materials and methods

### Reagents

Antibodies against eMyHC, myogenin, Pax7, and β3 integrin were purchased from Santa Cruz (Dallas, TX, USA). Antibodies against AKT, p-AKT, Myod1, PDIA1, PDIA3, and GAPDH were purchased from Abcam (Cambridge, UK). Antibodies against mTOR and P-mTOR were purchased from Cell Signaling Technology (Beverly, MA, USA). Antibodies against eMyHC were purchased from R&D (Minneapolis, MN, USA). The Cell Surface Protein Isolation Kit, Donkey anti-rabbit FITC, and donkey anti-mouse TRITC were purchased from Thermo Fisher Scientific (Waltham, MA USA). The ELISA kit for PDIA3 detection was obtained from R&D (Minneapolis, MN, USA). Cardiotoxin (CTX), LY294002, recombinant human Vitronectin and PDI inhibitors (16F16, EGCG, bacitracin, and PCAMA31) were obtained from Sigma-Aldrich (St. Louis, MO, USA).

### Animal model

Male C57 mice (6 weeks old, 20–25 g) were purchased from the Third Military Medical University Laboratory Animal Center. A total of 100 μl CTX (10 μM, dissolved in PBS) was injected into the gastrocnemius of mice to generate a model of muscle injury. At 3 and 7 days post-injury, the gastrocnemius was excised from euthanized mice to conduct the described analyses. In our study, all animal experimental protocols were approved by the Animal Care Committee of the Third Military Medical University according to the National Institutes of Health Guide for the Care and Use of Laboratory Animals (NIH publication number 8023, revised 1978).

### Myogenic cell culture

Murine C2C12 muscle cells were purchased from ATCC (Manassas, USA) and grown in high-glucose DMEM supplemented with 10% fetal bovine serum (HyClone, USA) and 1% penicillin/streptomycin at 37 °C in 5% CO_2_. Once they reached 70% confluence, C2C12 myoblasts were induced to differentiate for 48 h by adding DMEM containing 2% horse serum.

### Cell surface protein extraction

The Thermo Scientific Pierce Cell Surface Protein Isolation Kit (89881) enables the convenient biotinylation and isolation of cell surface proteins for Western blot analysis. Cells are first labeled with Thermo Scientific EZ-Link Sulfo-NHS-SS-Biotin, a thiol-cleavable amine-reactive biotinylation reagent. Cells are subsequently lysed with a mild detergent, and the labeled proteins are then isolated with Thermo Scientific NeutrAvidin Agarose. The bound proteins are released by incubating the lysates with SDS–PAGE sample buffer containing 50 mM DTT.

### Immunoblotting and coimmunoprecipitation assays

Immunoblotting assays were performed as described previously (4). Briefly, the proteins extracted from muscle and C2C12 cells were separated by SDS–PAGE and transferred to PVDF membranes. These membranes were incubated at 4 °C overnight with anti-PDIA1 (1:500), anti-PDIA3 (1:500), anti-eMyHC (1:200), anti-myogenin (1:1000), anti-Pax7 (1:1000), anti-Myod1 (1:1000) anti-eMyHC (1:500), anti-AKT (1:2000), anti-p-AKT (1:2000), anti-mTOR (1:500), anti-p-mTOR (1:500), anti-β3 integrin (1:500), and GAPDH (1:2500) antibodies, followed by three washes with TBST and incubation with the associated secondary antibodies at room temperature (RT) for 60 min. Following washing with TBST three times, the proteins of interest were detected with ECL reagents. The total proteins were immunoprecipitated with an anti-PDIA3 antibody and then immunoblotted with an anti-β3 integrin antibody.

### q-PCR

RNA was isolated from muscle and C2C12 cells using TRIzol (Invitrogen, USA). This RNA was used as a template for cDNA synthesis using reverse transcriptase (Invitrogen, USA). qPCR was performed with a sequence detection system (Bio-Rad, USA) with the following primers (Invitrogen, USA): eMyHC (forward, reverse) primers 5′-TCAGGATTCGGAGGAGCAGG-3′ and 5′-CTTCTTGTCCAGAGCGGCAG-3′; myogenin (forward, reverse) primers 5′-GGTCCCAACCCAGGAGATCA-3′ and 5′-CGTCTGGGAAGGCAACAGAC-3′; Myod1 (forward, reverse) primers 5′-ATGGCTACGACACCGCCTAC-3′ and 5′-AGATGCGCTCCACTATGCTG-3′; PDIA1 (forward, reverse) primers 5′-CTGGCAGCAGAGGCTATTGA-3′ and 5′-AAGTTGTTGCGGCCTTCATC-3′; PDIA3 (forward, reverse) primers 5′-ATGGATGCCACAGCCAATG-3′ and 5′-TCACGGCCACCTTCATACTTC-3′; GAPDH (forward, reverse) primers 5′-CTGCGACCACCAACTGCTTAGC-3′ and 5′-CTTCACCACCTTCTTGATGTC-3′.

### Immunofluorescence

Frozen sections of excised gastrocnemius tissue were fixed with acetone at 4 °C for 10 min, permeated with 0.03% Triton X-100 at RT for 30 min, and blocked with 2% BSA in PBS at RT for 60 min. Immunofluorescence staining of the muscle sections was carried out using primary antibodies against Pax7, myogenin, eMyHC, PDIA3, P-AKT, and β3 integrin at 4 °C overnight, and then the sections were incubated with the associated secondary antibodies (donkey anti-mouse TRITC or donkey anti-rabbit FITC) at RT for 1 h. Cells were seeded on rat tail collagen I (Invitrogen, USA)-coated coverslips for the differentiation analysis. At 1 and 2 days after differentiation, C2C12 cells were fixed with 4% polyoxymethylene at RT for 15 min and blocked with 2% BSA at RT for 30 min. The C2C12 cells were then incubated with primary antibodies against PDIA3 (1:1000) at 4 °C for 1 h, followed by incubation with the associated secondary antibodies for 1 h at 37 °C. The cell nuclei in muscle and C2C12 cells were labeled by DAPI (blue). All images were acquired and analyzed using a Zeiss LSM 720 confocal microscope.

### Histology

Paraffin-embedded and H&E-stained gastrocnemius sections were generated by the Third Military Medical University Biomedical Analysis Center.

### Statistical analysis

All experimental data are presented as the mean ± standard error of the mean. The statistical analysis of these data was assessed for significance using Student’s *t* test and one-way analysis of variance. The results with *P* values of <0.05 were considered to be significant. All data were analyzed with SPSS software (version 17.0).

## Results

### Upregulation of PDI in regenerated skeletal muscle

**Fig. 1:**
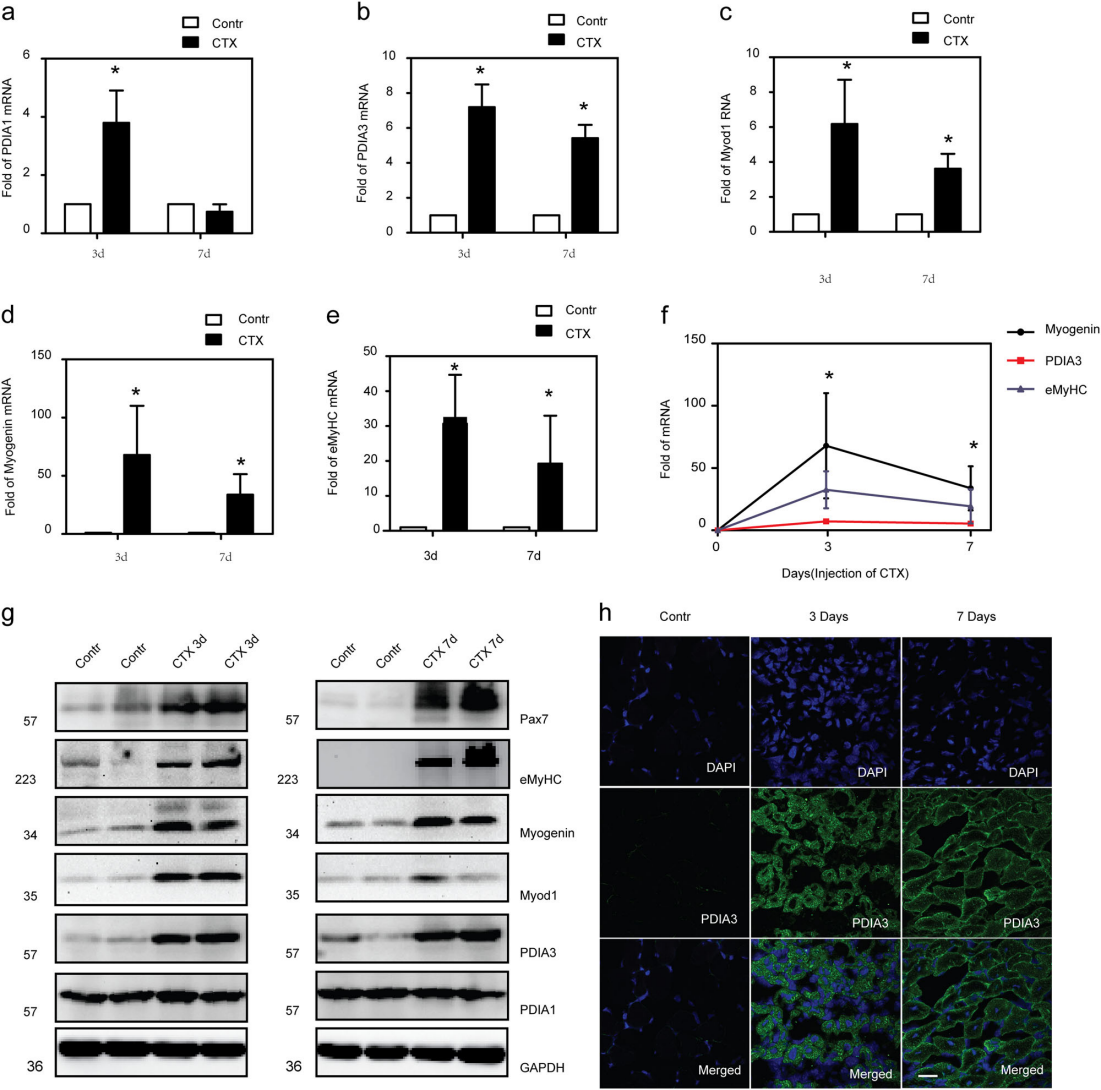
In vivo analysis of PDI expression in muscles in the CTX-induced injury model. The gastrocnemius muscle in mice was injured by the direct injection of 100 µl CTX (10 µM). On days 3 and 7 post injury, the gastrocnemius was harvested from euthanized mice. **a**–**f** The mRNA levels of PDIA1, PDIA3, Myod1, myogenin, and eMyHC in gastrocnemius muscle were tested by q-PCR (*P < 0.05 vs. Control; *n* = 4). **g** Protein abundances of PDIA1, PDIA3, Pax7, Myod1, myogenin, and eMyHC were measured by immunoblotting using antibodies (*n* = 4). **h** Frozen sections of gastrocnemius muscle were subjected to immunofluorescence staining of PDIA3. The cell nuclei were stained with DAPI. Scale 20 µm.

**Fig. 2:**
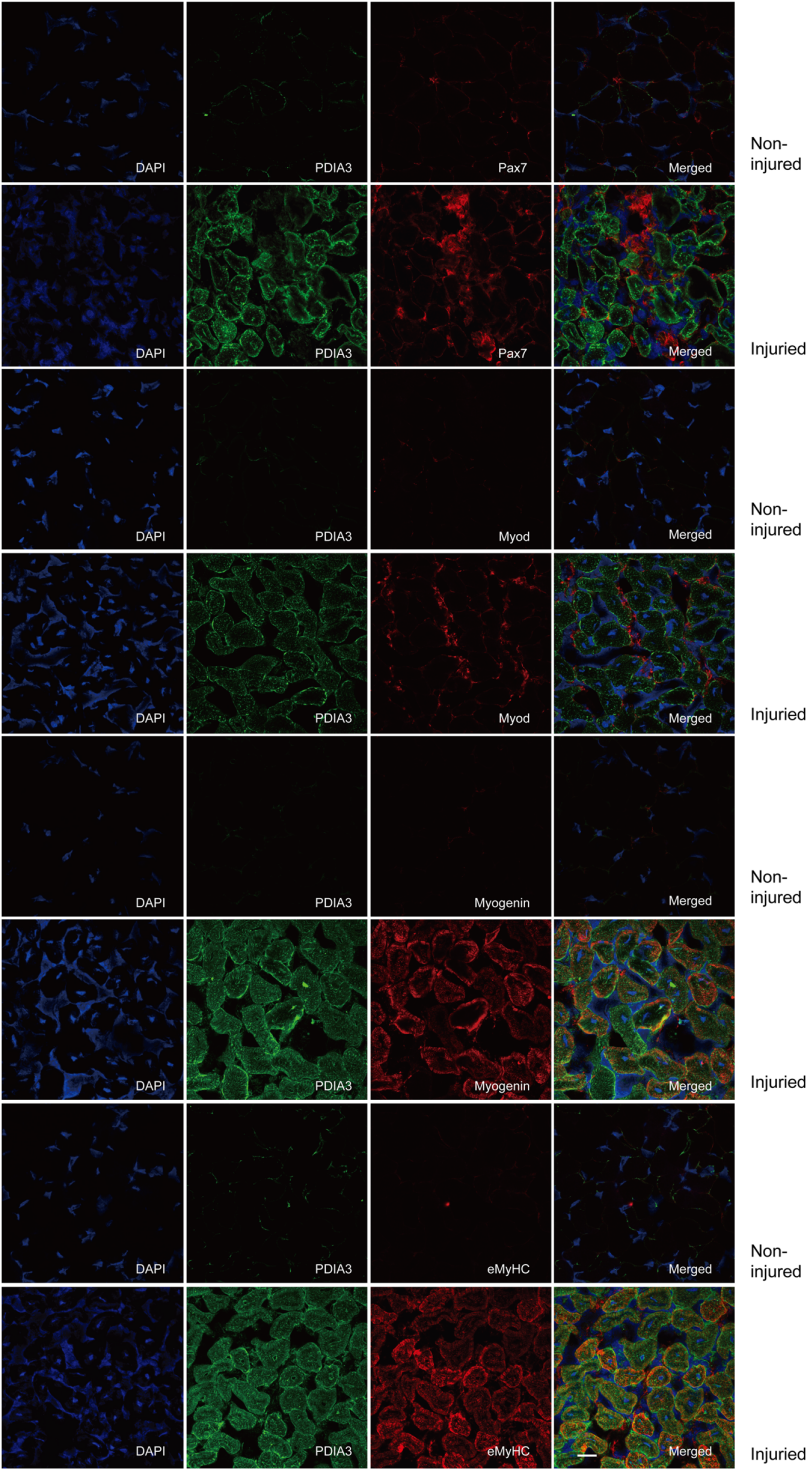
Expression of PDIA3 in activated SCs during muscle regeneration. The gastrocnemius muscle in mice was injured by the direct injection of 100 µl CTX (10 µM). On day 7 post injury, the gastrocnemius muscle was harvested from euthanized mice. Frozen sections of gastrocnemius muscle were subjected to the immunofluorescence costaining of PDIA3 with Pax7, Myod1, myogenin, and eMyHC. The staining of Pax7 was performed to identify SCs; the staining of Myod1 was used to identify activated SCs that had undergone differentiation. The staining of myogenin was performed to identify mature myotubes. The staining of eMyHC was used to identify regenerated myofibers. The cell nuclei were stained with DAPI. Scale 20 µm.

To test whether PDI participated in the regulation of skeletal muscle regeneration, we first measured the mRNA and protein levels of PDIA1 and PDIA3 in regenerated adult mouse skeletal muscle in the CTX injury model. As illustrated in Fig. 1a, the level of PDIA1 mRNA was increased on day 3 postinjury and dramatically downregulated to a normal level on day 7 postinjury. PDIA1 protein was expressed in uninjured muscles and was not altered at 3 days and 7 days after injury (Fig. 1g). In contrast, the level of PDIA3 mRNA was significantly increased on days 3 and 7 postinjury (Fig. 1b). Although PDIA3 was hardly detected in normal muscles, a significant increase in this protein was observed at 3 days and 7 days after injury (Fig. 1g). The immunofluorescence staining also showed that PDIA3 was induced in regenerated myofibers with centralized nuclei (Fig. 1h). It was notable that PDIA3 expression was upregulated and accompanied by the expression of myogenic markers, such as Pax7, Myod1, myogenin, and embryonic MyHC (eMyHC) (Fig. 1c–g). To further elucidate the role and mechanism of PDIA3 in myogenesis during muscle regeneration, immunofluorescence staining of PDIA3 and myogenic protein markers were simultaneously performed on frozen sections of skeletal muscles after injury induced by CTX. PDIA3 was hardly observed in activated SCs that were stained by Pax7 and Myod1 (Fig. 2) but was detected in neonatal myofibers that were myogenin- and eMyHC-positive (Fig. 2). These results suggest that PDIA3 plays a major role in terminal myogenesis during muscle regeneration.

### Inhibition of PDI activity attenuates muscle regeneration

**Fig. 3:**
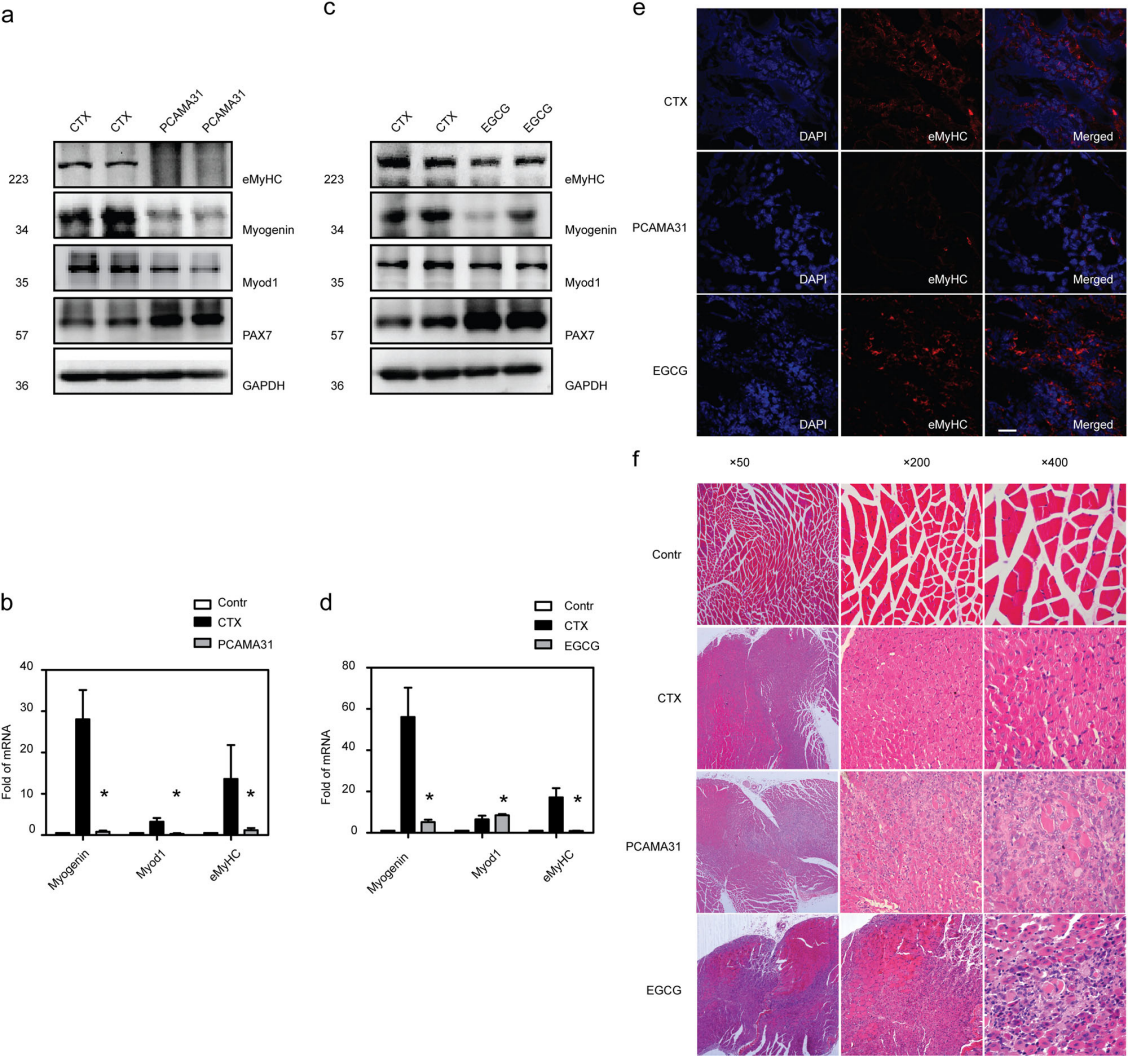
Inhibition of PDIA3 impaired muscle regeneration. The gastrocnemius was injured by the direct injection of 100 µl CTX (10 µM) and treated with PCAMA31 or EGCG. The gastrocnemius was collected 3 days after injury. **a** The levels of Pax7, Myod1, myogenin, and eMyHC in gastrocnemius muscle treated with PCAMA31 were analyzed by immunoblotting (*n* = 4). **b** The mRNA levels of Myod1, myogenin, and eMyHC in gastrocnemius muscle treated with PCAMA31 were tested by q-PCR (*P < 0.05 vs. Control; *n* = 4). **c** The levels of Pax7, Myod1, myogenin, and eMyHC in gastrocnemius muscle treated with EGCG were analyzed by immunoblotting (*n* = 4). **d** The mRNA levels of Myod1, myogenin, and eMyHC in gastrocnemius muscle treated with EGCG were tested by q-PCR (*P < 0.05 vs. Control; *n* = 4). **e** Frozen sections of gastrocnemius muscle were subjected to the immunofluorescence staining of eMyHC. The cell nuclei were stained with DAPI. The gastrocnemius was injured by the direct injection of 100 µl CTX (10 µM) and treated with PCAMA31 or EGCG. Scale 100 µm. **f** The gastrocnemius was collected 14 days after injury, and paraffin-embedded muscle sections were subjected to H&E staining.

To evaluate the role of PDIA3 in muscle regeneration in vivo, we tested whether the inhibition of PDIA3 impaired myogenesis and muscle regeneration in mice. At 3 days after CTX-induced injury, when myogenesis occurred, the expression of Myod1, myogenin, and eMyHC were decreased after administration of a PDI inhibitor (PCAMA31) (Fig. 3a, b). However, Pax7 expression was increased in PCAMA31-treated mice with injured muscles (Fig. 3a). In addition, the inhibition of PDIA3 by EGCG significantly increased Pax7 expression, and decreased myogenin and eMyHC expressions but had no effect on Myod1 expression (Fig. 3c, d). As shown in Fig. 3e, the amount of eMyHC expressed in regenerated myofibers was significantly reduced in both PCAMA31-treated and EGCG-treated mice compared with that in control muscles. Thus, PDIA3 appears to be essential for myogenic terminal differentiation. Finally, on day 14 after injury, regenerated myofibers with centralized nuclei predominated muscles in control mice but not those in PCAMA31-treated and EGCG-treated mice (Fig. 3f). Moreover, in treated mice, a pathologically high level of local inflammation was present, indicating the impairment of skeletal muscle regeneration (Fig. 3f). These data suggest that PDIA3 is critical to terminal differentiation during skeletal muscle regeneration.

### The role of PDIA3 in myoblast differentiation and fusion

**Fig. 4:**
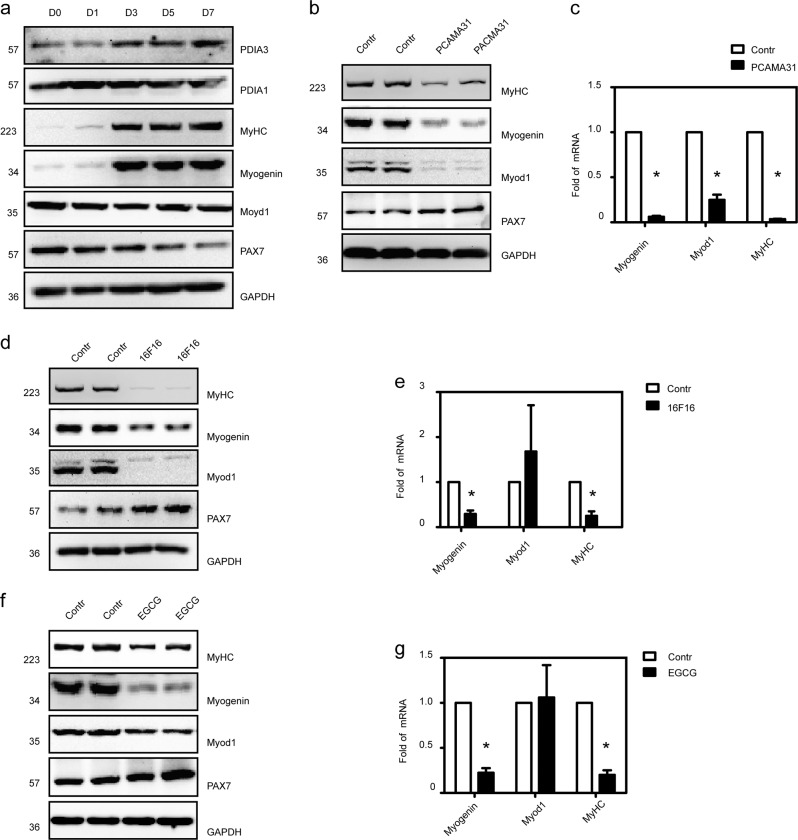
The role of PDIA3 in myoblast differentiation. **a** C2C12 myoblasts were induced to differentiate for 1, 3, 5, and 7 days. The expression of PDIA1, PDIA3, Pax7, myogenin, Myod1, and MyHC were detected by immunoblotting (*n* = 4). **b** C2C12 myoblasts were differentiated for 48 h and treated with PCAMA31. After 24 h, the expression of myogenin, Myod1, and MyHC were measured by immunoblotting (*n* = 4). **c** The levels of myogenin, Myod1, and MyHC mRNA in C2C12 cells treated with PCAMA31 were detected by q-PCR (*P < 0.05 vs. Control; *n* = 4). **d** C2C12 myoblasts were induced to differentiate for 48 h and treated with 16F16. After 24 h, the expression of myogenin, Myod1, and MyHC were assessed by immunoblotting (*n* = 4). **e** The levels of myogenin, Myod1, and MyHC mRNA in C2C12 cells treated with 16F16 were detected by q-PCR (*P < 0.05 vs. Control; *n* = 4). **f** C2C12 myoblasts were induced to differentiate for 48 h and treated with EGCG. After 24 h, the expression of myogenin, Myod1, and MyHC were tested by immunoblotting (*n* = 4). **g** The levels of myogenin, Myod1, and MyHC mRNA in C2C12 cells treated with EGCG were detected by q-PCR (*P < 0.05 vs. Control; *n* = 4).

To definitively confirm that PDIA3 functions in myogenesis, we employed multiple in vitro differentiation assays using C2C12 myoblasts, which are most frequently used to study the mechanisms of myogenic differentiation. Immunoblotting showed that both PDIA1 and PDIA3 were expressed in undifferentiated myoblasts and that PDIA3 expression was correlated with the levels of the myogenic marker proteins myogenin and MyHC, which increased gradually during C2C12 myoblast differentiation. However, the expression of PDIA1 was unaltered (Fig. 4a).

**Fig. 5:**
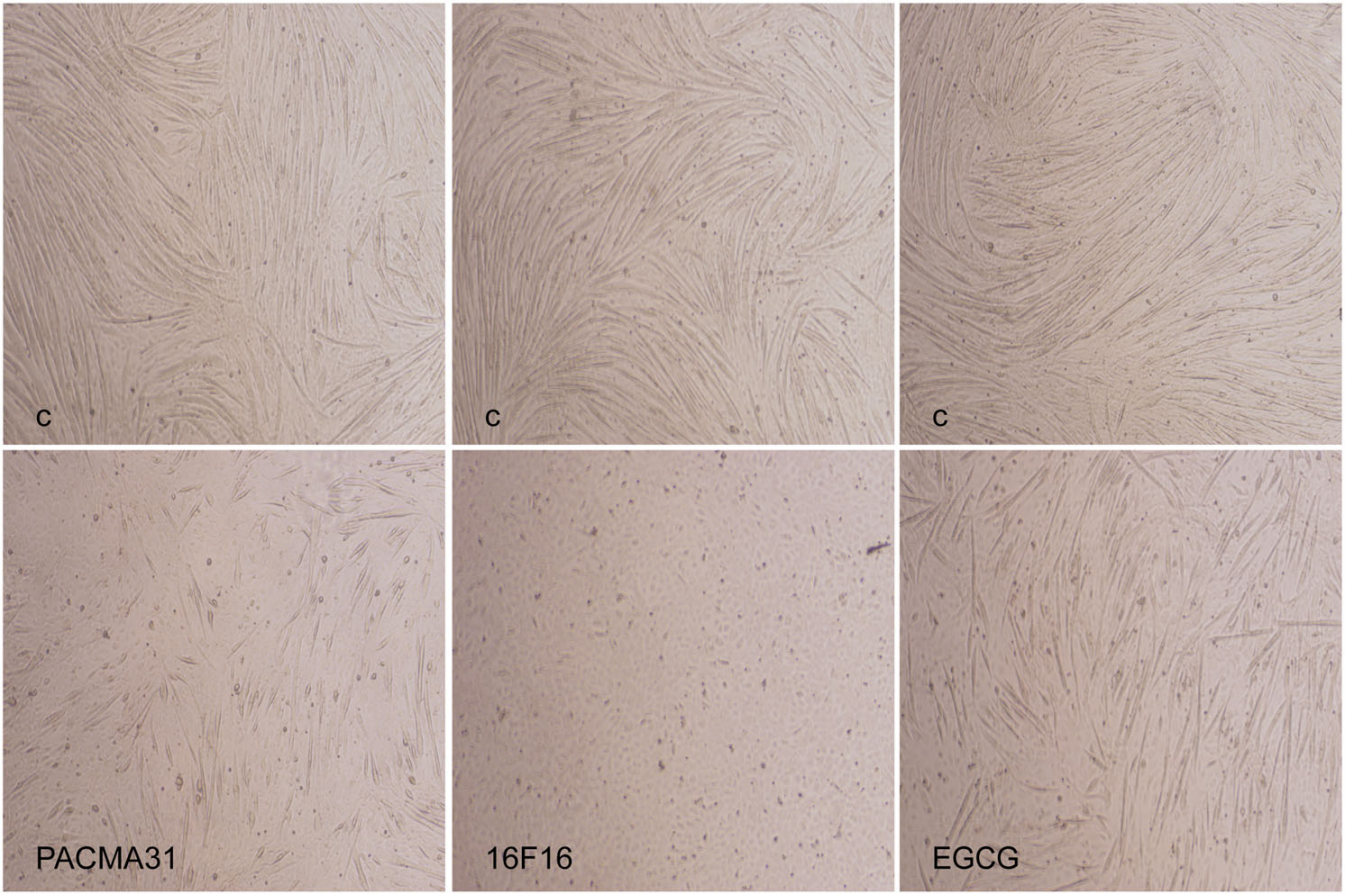
The role of PDIA3 in myoblast fusion. C2C12 myoblasts were induced to differentiate for 48 h and were treated with PCAMA31, 16F16, and EGCG. After 72 h, myotube formation was observed by a microscope. Scale 200 µm.

To further test whether PDIA3 mediated myoblast differentiation, we inhibited PDI activity by utilizing PDI inhibitors in C2C12 myoblasts. We initially examined the effect of PCAMA31 on myogenic marker expression and the fusion of C2C12 myoblasts. As shown in Figs. 4b, c and 5, PCAMA31 significantly decreased Myod1, myogenin, and MyHC expression and inhibited the fusion of myoblasts. In the myoblast differentiation experiment, when treated with PDI (16F16), which is a widely used PDI inhibitor, myoblasts failed to express Myod1, myogenin, and MyHC or to fuse into myotubes (Figs. 4d, e and 5). To confirm the involvement of PDIA3 in myogenesis, we tested the myogenic expression and fusion of C2C12 myoblasts by inhibiting PDIA3 using EGCG^24^. The results in Figs. 4f, g and 5 show that EGCG had no influence on Myod1 expression but decreased myogenin and MyHC expression and inhibited the fusion of myoblasts. These results indicate that a wide range of PDI inhibitors can interfere with the capacity for myogenesis, and suggest that PDIA3 is a major PDI isoform that mediates the capacity for myogenesis.

Taken together, the results of these experiments show that the PDI isoform PDIA3 plays a major role in mediating myogenesis, whereas the PDI isoform PDIA1 plays little or no role in this process.

### Extracellular PDIA3 is crucial for myoblast differentiation and fusion

**Fig. 6:**
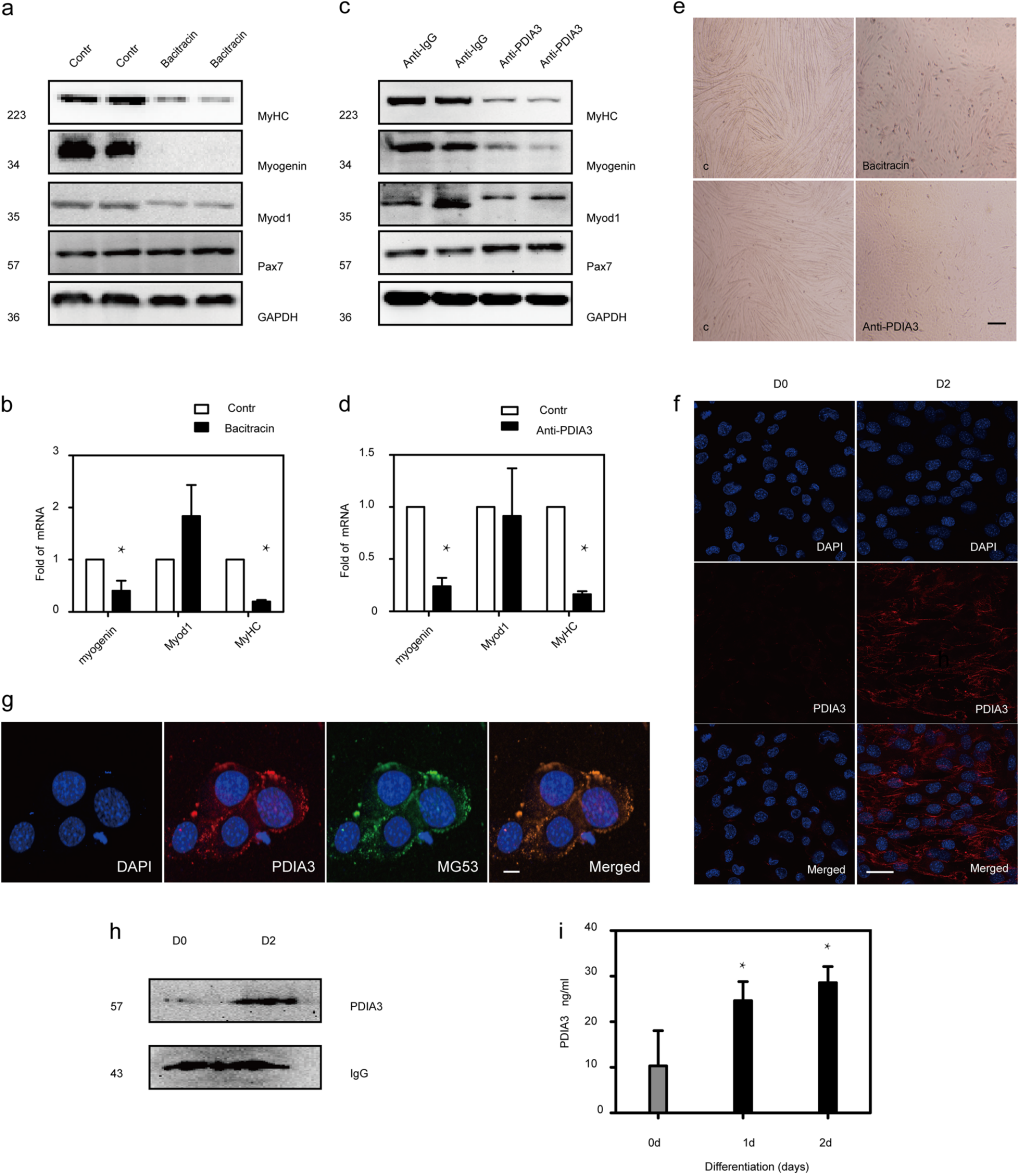
The role of extracellular PDIA3 in myoblast differentiation and fusion. **a**, **b** C2C12 myoblasts were induced to differentiate for 48 h and treated with bacitracin. After 24 h, the myogenin, Myod1, and MyHC levels were measured by immunoblotting and PCR (*P < 0.05 vs. Control; *n* = 4). **c**, **d** C2C12 myoblasts were induced to differentiate for 48 h and treated with a monoclonal anti-PDIA3 antibody or anti-IgG (10 µg/ml). After 24 h, the myogenin, Myod1, and MyHC levels were measured by immunoblotting and PCR (*P < 0.05 vs. Control; *n* = 4). **e** C2C12 myoblasts were induced to differentiate for 48 h and treated with bacitracin and a monoclonal anti-PDIA3 antibody. Myotube formation was observed by a microscope. Scale 200 µm. **f** C2C12 myoblasts were differentiated for 48 h and then subjected to the live imaging of PDIA3. Scale 40 µm. **g** C2C12 myoblasts were differentiated for 48 h and then subjected to the live imaging of PDIA3 and MG53. Scale 40 µm. **h** C2C12 myoblasts were induced to differentiate for 48 h. The level of PDIA3 on the cell membrane was measured by immunoblotting. **i** C2C12 myoblasts were induced to differentiate for 24 and 48 h. The level of PDIA3 in the cellular supernatant was measured by using an anti-PDIA3 ELISA kit (*P < 0.05 vs. Control; *n* = 4).

To investigate whether extracellular PDIA3 is involved in myoblast differentiation and fusion, we first analyzed the effects of the membrane impermeable inhibitor bacitracin on myogenic marker expression and the fusion of myoblasts. When C2C12 myoblasts were induced to differentiate in the presence of bacitracin, there were significant reductions in the amounts of the proteins Myod1, myogenin, and MyHC, and the numbers of myotubes with respect to those in control cells (Fig. 6a, b, e). In addition to bacitracin, we performed C2C12 differentiation in the presence of a blocking monoclonal anti-PDIA3 antibody. We found that the anti-PDIA3 antibody significantly decreased the number of myotubes and inhibited myogenin and MyHC expression but did not alter Myod1 expression (Fig. 6c–e).

Because the evidence from the inhibition studies suggested that there was a critical effect of extracellular PDIA3 activity on myoblast differentiation, we used immunofluorescence staining analysis to detect the presence of PDIA3 on myoblasts and myotube membrane surfaces. Live cell staining showed that PDIA3 was expressed on differentiated myotube membrane surfaces but not on undifferentiated myoblast membranes (Fig. 6f). Additionally, live cell staining showed that PDIA3 was co-localized on myotube membranes with the membrane protein marker MG53 (Fig. 6g). We further tested the expression of PDIA3 on membranes by immunoblotting. As Fig. 6h illustrates, PDIA3 was upregulated on differentiated myotube membranes compared with undifferentiated myoblast membranes. In addition, we used an ELISA assay to measure the level of PDIA3 protein in the extracellular medium of C2C12 cells before differentiation and after differentiation. PDIA3 protein was present in the extracellular medium, and a dramatic increase in this protein was observed at 1 and 2 days after differentiation (Fig. 6i).

Thus, these results indicate that PDIA3 is secreted during C2C12 differentiation, and this autocrine signaling by PDIA3 is crucial to myoblast differentiation and fusion.

### Extracellular PDIA3 regulates myoblast differentiation and fusion via AKT/mTOR signaling

**Fig. 7:**
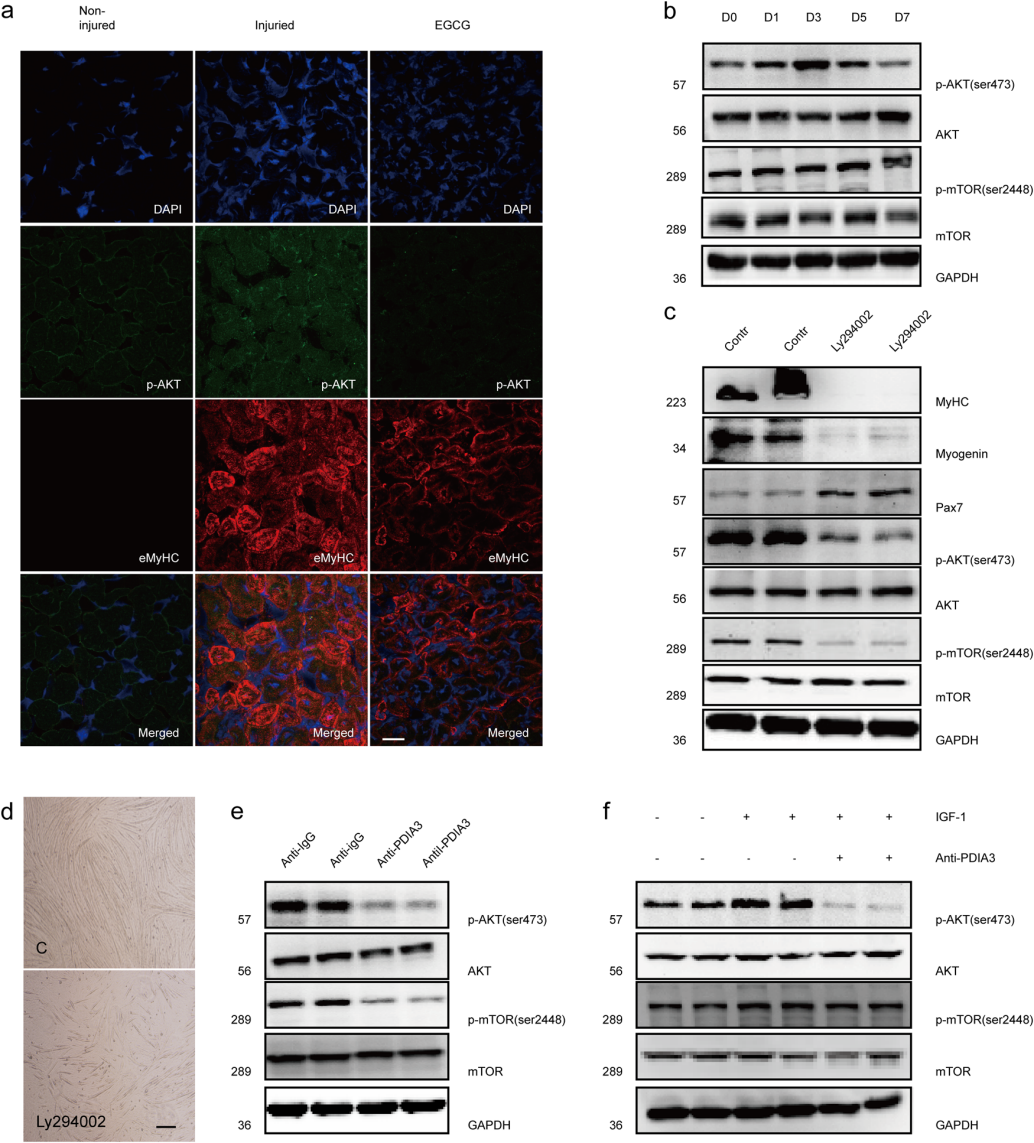
PDIA3 regulates myoblast differentiation through AKT/mTOR signaling. **a** The gastrocnemius in mice was injured by the direct injection of 100 µl CTX (10 µM) and the intraperitoneal injection of EGCG. On day 7 post injury, the gastrocnemius was harvested from euthanized mice. Frozen sections of gastrocnemius muscle were subjected to the immunofluorescence staining of p-AKT and eMyHC. Scale 20 µm. **b** C2C12 myoblasts were induced to differentiate for 1, 3, 5, and 7 days. The levels of the p-AKT, AKT, p-mTOR, and mTOR proteins were assessed by immunoblotting (*n* = 4). **c** C2C12 myoblasts were induced to differentiate for 48 h and treated with LY90042. After 24 h, the levels of the MyHC, myogenin, p-AKT, AKT, p-mTOR, and mTOR proteins were assessed by immunoblotting (*n* = 4). **d** C2C12 myoblasts were induced to differentiate for 48 h and treated with LY90042. After 72 h, myotube formation was observed by a microscope. Scale 200 µm. **e** C2C12 myoblasts were induced to differentiate for 48 h, followed by treatment with an anti-PDIA3 antibody for 24 h. The expression of p-AKT, AKT, p-mTOR, and mTOR were measured by immunoblotting (*n* = 4). **f** C2C12 myoblasts were induced to differentiate for 48 h and treated with IGF-1 (2 µg/ml) for 24 h in the absence or presence of anti-PDIA3 (10 µg/ml) antibody. The levels of the p-AKT, AKT, p-mTOR, and mTOR proteins were measured by immunoblotting (*n* = 4).

To assess the time-course of AKT/mTOR pathway activity in the CTX injury model, we first examined the phosphorylation levels of AKT (p-AKT) in muscle after injury. Accompanied by the induction of MyHC expression, the level of p-AKT was significantly increased in regenerated myofibers (Fig. 7a). In vitro, the levels of P-AKT and P-mTOR were both increased during C2C12 myoblast differentiation, while the total AKT and mTOR levels were not altered (Fig. 7b). Moreover, we induced C2C12 myoblast differentiation in the presence of LY294002, a compound that inhibits AKT/mTOR signaling. As shown in Fig. 7c, d, LY294002 significantly decreased the expression of p-AKT, p-mTOR, myogenin, and MyHC and blocked myoblast fusion. These results confirm that AKT/mTOR signaling is critical for myogenesis and muscle regeneration.

To determine whether PDIA3 regulates myoblast differentiation and fusion via AKT/mTOR signaling, we first showed that the inhibition of PDIA3 significantly reduced p-AKT and eMyHC expression in CTX-damaged muscle (Fig. 7a). In vitro, p-AKT and p-mTOR expression were also inhibited by anti-PDIA3 antibody during myoblast differentiation (Fig. 7e). IGF-1, an essential factor in myoblast differentiation, clearly increased the levels of p-AKT and p-mTOR; however, these effects were diminished by the anti-PDIA3 antibody (Fig. 7f).

### PDIA3-mediated myoblast differentiation and fusion depends on β3 integrin

**Fig. 8:**
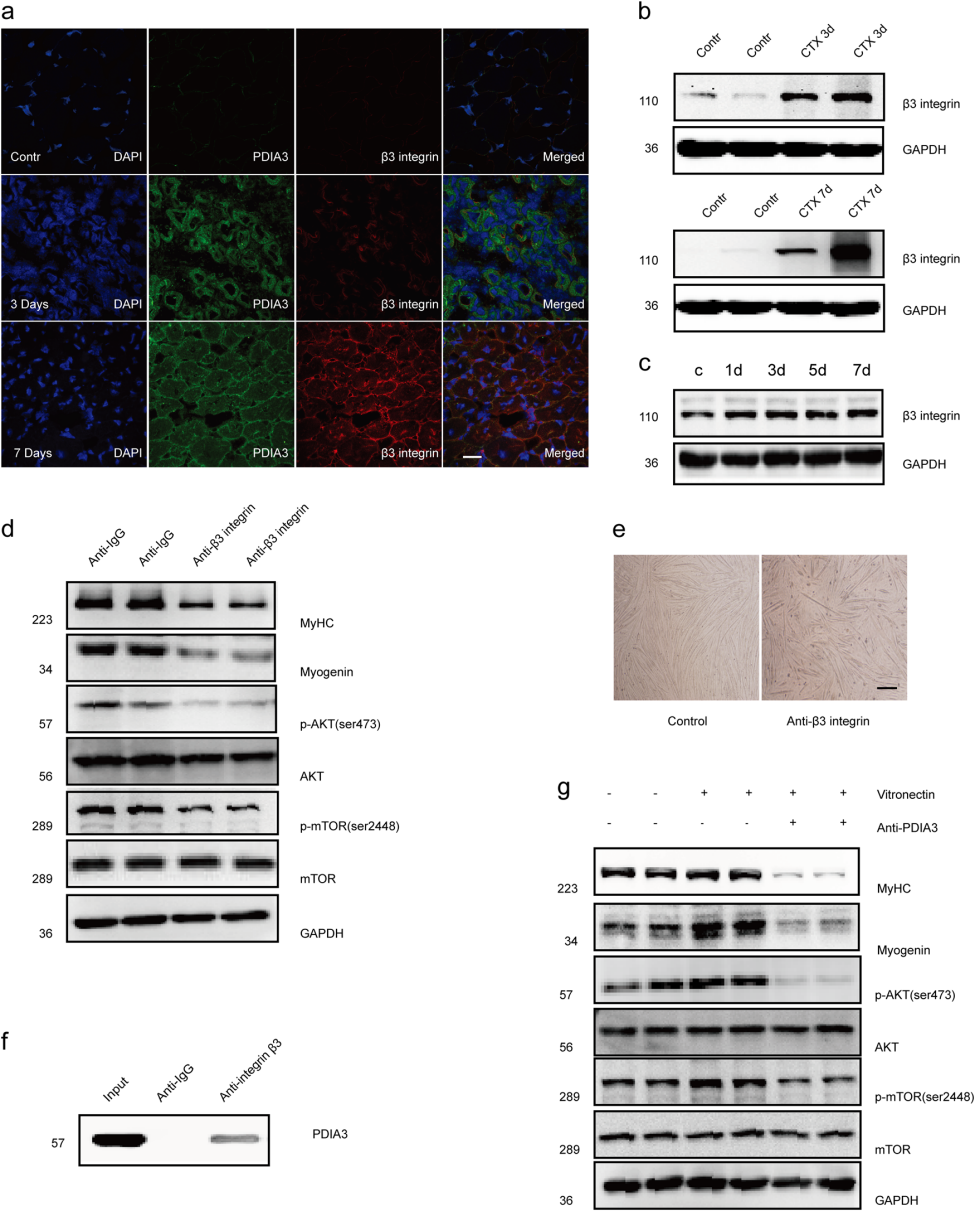
PDIA3 regulates β3 integrin-mediated myoblast differentiation and fusion. **a** Frozen sections of gastrocnemius muscle were subjected to the immunofluorescence costaining of β3 integrin and PDIA3. Scale 20 µm. **b** The gastrocnemius in mice were injured by the direct injection of 100 µl CTX (10 µM) and collected at 3 and 7 days after injury. The level of β3 integrin protein was measured by immunoblotting. (*n* = 4). **c** C2C12 myoblasts were subjected to differentiation for 1, 3, 5, and 7 days. The β3 integrin levels were measured by immunoblotting (*n* = 4). **d** C2C12 myoblasts were induced to differentiate for 48 h, followed by monoclonal anti-β3 integrin (10 µg/ml) treatment for 24 h. The levels of p-AKT, AKT, p-mTOR, mTOR, myogenin, and MyHC were measured by immunoblotting (*n* = 4). **e** C2C12 myoblasts were induced to differentiate for 48 h, followed by monoclonal anti-β3 integrin (10 µg/ml) treatment for 72 h. Myotube formation was observed by a microscope. Scale 200 µm. **f** The gastrocnemius in mice were injured by the direct injection of 100 µl CTX (10 µM) and collected at 7 day after injury. The muscle lysates were subjected to immunoprecipitation with an anti-PDIA3 antibody and were immunoblotted with anti-β3 integrin antibody (*n* = 4). **g** C2C12 myoblasts were induced to differentiate for 48 h and incubated with recombinant human vitronectin (2 µg/ml) for 24 h in the absence or presence of an anti-PDIA3 (10 µg/ml) antibody. The levels of p-AKT, AKT, p-mTOR, mTOR, myogenin, and MyHC were measured by immunoblotting (*n* = 4).

To test whether PDIA3 mediation of myogenesis depended on β3 integrin, we first detected the expression of β3 integrin in regenerated muscles using the CTX injury model. Immunofluorescence staining revealed that β3 integrin expression was induced in regenerated myofibers with centralized nuclei (Fig. 8a). Similar to PDIA3, β3 integrin was undetected in uninjured muscles but was expressed in injured muscle at 3–7 days after injury (Fig. 8b). An in vitro study showed that β3 integrin was upregulated during myogenic differentiation (Fig. 8c). We further induced myoblast differentiation by blocking β3 integrin using a monoclonal anti-β3 integrin antibody. As Fig. 8d, e show, the anti-β3 integrin antibody significantly decreased p-AKT, P-mTOR, myogenin, and MyHC expression, ultimately inhibiting myoblast fusion. These results indicate that β3 integrin is a major component that facilitates myogenesis and muscle regeneration through the AKT/mTOR pathway.

Because the evidence obtained from the immunofluorescence staining suggested that PDIA3 colocalized with β3 integrin in the muscle membrane in neonatal mice (Fig. 8a), we performed immunoprecipitation assays using lysates derived from differentiated myoblasts. We found that PDIA3 was coimmunoprecipitated with β3 integrin (Fig. 8f). We further carried out myoblast differentiation by activating β3 integrin using recombinant vitronectin, which is a widely used ligand of β3 integrin. Vitronectin treatment significantly enhanced myogenin and MyHC expression; however, these effects were diminished by the anti-PDIA3 antibody (Fig. 8g). This observation suggested that PDIA3 regulated β3 integrin-mediated myoblast differentiation via the AKT/mTOR-signaling pathway.

## Discussion

The current study demonstrates that extracellular PDIA3 plays a critical role in regulating myoblast differentiation and fusion during muscle regeneration. Furthermore, our results reveal that extracellular PDIA3 is associated with activated β3 integrin and AKT/mTOR signaling in myogenesis during muscle regeneration. Thus, we report the regulatory role of PDIA3 in muscle regeneration, which is mainly mediated by β3 integrin, in myoblast differentiation and fusion via AKT/mTOR signaling.

During muscle regeneration, myogenesis is tightly regulated by a combination of signaling molecules that successively induces the expression of myogenic transcription factors (such as Pax7, Myod1, and myogenin) to control SC self-renewal and differentiation^25^. We demonstrated here that PDIA3 expression was induced in mature regenerated myofibers (myogenin-positive and MyHC-positive) but was undetected in activated SCs (Pax7-positive and Myod1-positive), indicating a physiological role of PDIA3 in terminal myogenic differentiation rather than the earlier initiation of myogenesis. The in vivo studies further revealed that the inhibition of PDIA3 by EGCG could suppress myoblast terminal differentiation and fusion. However, we still need to consider the potential effects of EGCG on the activity of members of the PDI family, such as PDIA1 and PDIA224.

Interestingly, we have shown that PDIA3 accumulates on the membrane in regenerated muscle, which is also detected outside the cell during myoblast differentiation in vitro. The specific inhibition of extracellular PDIA3 activity with monoclonal anti-PDIA3 antibody significantly inhibits myogenic terminal differentiation and the fusion of myoblasts. Thus, it appears that extracellular PDIA3 is required for myogenic terminal differentiation and the fusion of myoblasts in the late phase of muscle regeneration.

Although a previous study reported that extracellular PDIA3 could directly bind to β3 integrin to regulate αvβ3 integrin-mediated coagulation, neutrophil migration, T cell migration, and sperm–egg fusion^19–23,26^, it was unknown whether this PDIA3-mediated β3 integrin activity was involved in myogenesis during muscle regeneration. Recently, β3 integrin has been reported to be transiently induced in activated SCs, which is essential for the initiation of myogenesis in SCs, and its deficiency disrupts myogenesis and impairs muscle regeneration^17^. In this study, we found that β3 integrin expression is also induced in regenerating myofibers and gradually increases in C2C12 myoblasts during differentiation and fusion. The administration of an anti-β3 integrin antibody significantly inhibited myogenic terminal differentiation and impaired myotube formation, suggesting that β3 integrin is also required for these two main steps of myogenesis. Furthermore, we demonstrated that PDIA3 directly bound to β3 integrin and that β3 integrin co-localized with PDIA3 in myofiber membranes. The positive effects of β3 integrin-mediated myoblast differentiation was diminished by the anti-PDIA3 antibody. Thus, we concluded that extracellular PDIA3 regulates β3 integrin-mediated myoblast differentiation and fusion.

To the best of our knowledge, the AKT/mTOR-associated signaling pathway is crucial for myogenic terminal differentiation and remodeling during muscle regeneration^27^. It has been established that muscle injury induces the expression of IGF-1 and further stimulates the AKT/mTOR-signaling pathway, which is beneficial for muscle regeneration^12,28^. Here, we observed the significant activation of AKT/mTOR signaling in regenerating myofibers. The blockade of myogenin and MyHC expression and myoblast fusion by LY294002 further supported the notion that AKT/mTOR signaling plays a key role in terminal myogenic differentiation and fusion. We further found that cell-surface PDIA3 and β3 integrin were two components involved in maintaining AKT/mTOR signaling during muscle regeneration. In addition, the effect of β3 integrin on AKT/mTOR signaling was diminished by the anti-PDIA3 antibody, indicating that β3 integrin-mediated AKT/mTOR signaling during myogenesis depends on extracellular PDIA3.

In summary, our findings have identified PDIA3 as an autocrine factor expressed specifically in regenerating myofibers that is required for the activation of β3 integrin and AKT/mTOR signaling during myoblast differentiation and fusion. However, considering the complexity and multifunctional activity of members of the PDI family, further study of the mechanism underlying the effects of other PDIs (such as PDIA1 and PDIA2) on myogenesis is warranted.
